# Diverse and Dynamic Alpha-Neurotoxicity Within Venoms from the Palearctic Viperid Snake Clade of *Daboia*, *Macrovipera*, *Montivipera*, and *Vipera*

**DOI:** 10.1007/s12640-022-00572-w

**Published:** 2022-10-04

**Authors:** Abhinandan Chowdhury, Christina N. Zdenek, Bryan G. Fry

**Affiliations:** 1 Venom Evolution Lab, School of Biological Science, University of Queensland, St. Lucia QLD, 4072 Australia; 2 Department of Biochemistry & Microbiology, North South University, Dhaka, 1229 Bangladesh

**Keywords:** Venom, Alpha-neurotoxicity, *Daboia*, *Macrovipera*, *Montivipera*, *Vipera*

## Abstract

The targeting of specific prey by snake venom toxins is a fascinating aspect of molecular and ecological evolution. Neurotoxic targeting by elapid snakes dominates the literature in this regard; however, recent studies have revealed viper toxins also induce neurotoxic effect. While this effect is thought to primarily be driven by prey selectivity, no study has quantified the taxonomically specific neurotoxicity of the viper clade consisting of *Daboia*, *Macrovipera*, *Montivipera*, and *Vipera* genera. Here, we tested venom toxin binding from 28 species of vipers from the four genera on the alpha 1 neuronal nicotinic acetylcholine receptors (nAChRs) orthosteric sites of amphibian, avian, lizard, rodent, and human mimotopes (synthetic peptides) using the Octet HTX biolayer interferometry platform. *Daboia siamensis* and *D. russelii* had broad binding affinity towards all mimotopes, while *D. palestinae* had selectivity toward lizard. *Macrovipera* species, on the other hand, were observed to have a higher affinity for amphibian mimotopes except for *M. schweizeri*, which inclined more toward lizard mimotopes. All *Montivipera* and most *Vipera* species also had higher affinity toward lizard mimotopes. *Vipera a. montandoni*, *V. latastei*, *V. nikolski*, and *V. transcaucasina* had the least binding to any of the mimotopes of the study. While a wide range of affinity binding towards various mimotopes were observed within the clade, the lowest affinity occurred towards the human target. *Daboia siamensis* and *Macrovipera lebetina* exhibited the greatest affinity toward the human mimotope, albeit still the least targeted of the mimotopes within those species. Overlaying this toxin-targeting trait over phylogeny of this clade revealed multiple cases of amplification of this trait and several cases of secondary loss. Overall, our results reveal dynamic variation, amplification, and some secondary loss of the prey targeting trait by alpha-neurotoxins within the venoms of this clade, indicating evolutionary selection pressure shaping the basic biochemistry of these venoms. Our work illustrates the successful use of this biophysical assay to further research snake venom neurotoxins and emphasizes the risk of generalizing venom effects observed on laboratory animals to have similar effects on humans.

## Introduction

Snake venoms can exert a diversity of pathophysiological effects on any part of the body reachable by the bloodstream. (Boyer et al. 2015). While snake venom toxins are considered to be too large to cross the blood–brain barrier and affect the central nervous system, they can produce deleterious and lethal effects by binding to neuronal targets in the peripheral nervous system. Within the peripheral nervous system and in the neuromuscular junction, toxins can disrupt neurological signals by binding to various sites on the nerves (presynaptic neurotoxicity) or sites on the muscle cells (postsynaptic neurotoxicity) (Fry et al. 2009). To aid in prey immobilization, there have been multiple, repeated convergent evolution events for the targeting of the same neurological sites by different toxins within the vast array of snake venoms (Casewell et al. 2013; Fry et al. 2009; Healy et al. 2019; Xie et al. 2022).

Presynaptic snake venoms act on a myriad of targets to produce pain and paralysis, including calcium channels, potassium channels, and sodium channels (Dashevsky et al. 2022, 2021; Eng et al. 2015; Fry et al. 2009; Sunagar et al. 2015a, b; Utkin et al. 2015; Yang et al. 2016). Presynaptic binding may lead to either spastic or flaccid paralysis. Flaccid paralysis due to presynaptic action is produced by the phospholipase A_2_ toxins that have independently evolved in elapids (Group I) and viperids (Group II), and in both cases, the exact mechanisms of actions have not been fully elucidated. However, it is clear that the presynaptic binding of toxins results in nerve terminals being physically damaged, thereby preventing the release of acetylcholine (Sunagar et al. 2015a, b). Flaccid paralysis is produced by venoms such as *Calliophis intestinalis* which blocks the opening of Na_V_1.4 sodium channels, thereby preventing the release of acetylcholine from the nerve terminals and thus also preventing the contraction of the corresponding skeletal muscle (Dashevsky et al. 2022). Other elapid snakes cause flaccid paralysis through postsynaptic binding (described below). In contrast, the related species *Calliophis bivirgatus* possesses toxins that delay the inactivation of Na_V_1.4 sodium channels, causing acetylcholine overdose that results in rapid and uncontrollable contraction of the corresponding muscle (Dashevsky et al. 2021; Yang et al. 2016). Spastic paralysis is also produced by toxins from venoms in the genus *Dendroaspis* (Mamba). These venoms exert their toxicity by blocking voltage-gated potassium channels, again resulting in sustained release of acetylcholine, similarly leading to spastic paralysis (Harvey and Karlsson 1980). Other additional *Dendroaspis* toxins act within the synaptic cleft to inhibit acetylcholinesterase, thereby extending acetylcholine half-life beyond that of the normal regulatory cycle parameters (Karlsson et al. 1984).

As with presynaptic toxins, postsynaptic toxins act upon diverse molecular sites (Fry et al. 2009). This includes the blockage of voltage-dependent L-type calcium channels (CaV) in the cardiac muscle, resulting in loss of contractility, which has evolved on at least three occasions within elapid snakes: once in *Oxyuranus* and twice within *Dendroaspis* venoms (de Weille et al. 1991; Possani et al. 1992). The most highly expressed types of postsynaptic neurotoxins are alpha-neurotoxins that bind to the alpha-1 subunit of the nicotinic acetylcholine receptor, thereby blocking the contraction of skeletal and other voluntary muscles such as the diaphragm (Fry et al. 2009). Systemic paralysis of skeletal muscles prevents prey from escaping or retaliation due to loss of mobility, a decrease in metabolic fitness from a drop in metabolic respiration, and ultimate death due to respiratory failure (Boyer et al. 2015; Fry et al. 2015a). The successful targeting of the postsynaptic region of the neuromuscular by snake venom toxins is evidenced by its convergent evolution on multiple occasions: 3FTx (three-finger toxins) that evolved at the base of the snake radiation within basal snakes such as *Cylindrophis* and *Eryx* (Utkin et al. 2015); waglerin peptides isolated from *Tropidolaemus* viperid snakes and Azemiopsin peptides from *Azemiopis* venoms (both of which evolved as de novo toxin types within the natriuretic gene propeptide region and may represent derivations of the same origin event) (Xie et al. 2022); and viperid snake phospholipase A_2_ toxins (Vulfius et al. 2011, 2014).

While a diversity of toxin types capable of neurotoxic activity have been characterized, there are significant knowledge gaps. This is largely due to the low-throughput nature of conventional screening methods and taxonomic limitation of organ-bath tissue preparations, with the chick biventer the most widely used, the rat phrenic nerve preparation much less so, and the use of frog tissue preparations reported only a handful of times in the literature (Fry et al. 2015b). Thus, the vast majority of venoms have not been screened for such activity, and of those which have been screened, most are from the elapid snake family and taxonomically limited. For the venoms which have been screened on more than one tissue type, extreme taxon-selectivity has been observed, as has been the case for venoms that have been tested for lethal effects on multiple taxa of live organisms (Heyborne and Mackessy 2013; Modahl et al. 2018; Pawlak et al. 2006; Pawlak et al. 2009). The use of tissue preparations is expensive and requires animal ethics approval for the necessary euthanasia to dissect out the tissues. Therefore, restricting its use to common, domestically raised species such as domestic chickens and laboratory rats. Lethal testing on live animals requires higher-level approval, which is often impossible to obtain or outright banned in many institutions. Furthermore, approvals are likely only to be given for testing on common or captive species, with human subjects obviously impossible. To obtain insights of toxin binding to human-specific receptors, extrapolations are made from testing on cloned receptors within frog oocytes, but this technique is cumbersome, expensive, and manually intensive.

A recent alternative innovation uses the peptidic mimotopes (synthetic peptides) of the alpha-1 orthosteric site on the Octet^®^ biolayer interferometry (BLI) (Sartorius, Germany) platform as a taxonomically diverse high-throughput method (Zdenek et al. 2019). This animal-free method has proven to be a successful replacement of the low-throughput methods described above that rely upon sacrificing animals or difficult cloning procedures (Fry et al. 2015b). This BLI method has successfully revealed taxon-selective neurotoxicity such as preferential binding to a snake target by the snake-feeding specialist *Ophiophagus hannah* (King Cobra), fish by sea snakes, and amphibians for cobras (Harris et al. 2020c; Zdenek et al. 2019). This method has also facilitated the characterization of neurotoxicity in species not previously tested for these effects, including the Asian pit viper species *Calloselasma rhodostoma*, species within the African viperid genus *Bitis*, and the Central American arboreal genus *Bothriechis* (Harris et al. 2020b; Youngman et al. 2021, 2022). Further garnered from the BLI method are the residues responsible for venom resistance that convergently evolved in diverse animals ranging from the Honey Badger (*Mellivora capensis*) to the Burmese Python (*Python bivittatus*) to the Savannah Monitor Lizard (*Varanus exanthematicus*) to primates (Harris and Fry 2021; Harris et al. 2021; Jones et al. 2021).

Within the wide-ranging Palearctic viperid snakes in the clade formed by the related genera *Daboia*, *Macrovipera*, *Montivipera*, and *Vipera*, significant neurotoxicity has been reported as a clinical feature in human envenomations by some species of *Daboia* (*D. russelii* and *D. siamensis*) and *Vipera* (*V. ammodytes* and *V. aspis*), and milder but still notable neurotoxicity from *M. lebetina* envenomation (Malina et al. 2013; Persson 2015; Ranawaka et al. 2013; Silva et al. 2017, 2016; Turkmen et al. 2015). Congruent with this, both presynaptic and postsynaptic neurotoxins within this clade have been characterized (Freedman and Snyder 1981; Georgieva et al. 2000; Jan et al. 2002; Kasturi and Gowda 1989; Latinovic et al. 2016; Ritonja and Gubensek 1985; Slater et al. 1983, 1985). However, research has been concentrated upon the *Daboia* and *Vipera* species that are known to produce human clinical effects, with testing only on taxonomically limited tissue preparations. Thus, there is a significant knowledge gap regarding not only the relative neurotoxicity across all four genera but for their effects upon lineages other than avian, rodent, or human.

To fill this knowledge gap, we tested the binding of mimotopes corresponding to the orthosteric sites of amphibian, lizard, avian, rodent, and human alpha-1 nicotinic acetylcholine receptors on the Octet HTX platform by venoms from the full taxonomical range of the Palearctic viperid snake clade: *D. mauritanica*, *D. palaestinae*, *D. russelii*, *D. siamensis; Macrovipera lebetina cernovi*, *M. lebetina obtusa*, *M. lebetina turanica*, *M. schweizeri; Montivipera albizona*, *M. bornmuelleri*, *M. bulgardaghica*, *M. latifii*, *M. raddei*, *M. wagneri*, *M. xanthina*,*;* and *Vipera ammodytes*, *V. ammodytes meridonalis*, *V. ammodytes montandoni*, *V. aspis*, *V. aspis hugyi*, *V. berus*, *V. kaznakovi*, *V. latastei*, *V. latastei gaditana*, *V. nikolskii*, *V. renardi*, and *V. transcaucasiana*. Our results provide evolutionary insights regarding venom neurotoxicity diversity and also the potential for human effects due to binding to this particular neuropathological target.

## Materials and Methods

### Venom Collection and Preparation

All the venom study protocols of this work were performed with the approval of the University of Queensland Biosafety Approval #IBC134BSBS2015 and University of Queensland Animal Ethics approval 2021/AE00007. Lyophilized venoms (pooled venoms N = 3 adults) were reconstituted to 1 mg/ml working stock by adding 50% glycerol and deionized water and stored at − 20 °C for further use. The concentration was determined using 280-nm wavelength on a NanoDrop 2000 UV–Vis Spectrophotometer (Thermo Fisher Scientific™, Sydney, Australia). The Paleartic vipers (location if known) included in the study (obtained from the licensed venom supplier Latoxan, from captive snakes with the founding locality of the stock noted): *Daboia mauritanica* 1 (Tunisian population formerly considered as *D. deserti* (Martínez-Freiría et al. 2017) and referred to as *D. deserti* in a previous study on the coagulotoxicity actions (Chowdhury et al. 2021)), *Daboia mauritanica* 2 (Morocco), *Daboia palaestinae*, *Daboia russelii* (Pakistan), *D. siamensis* (Java), *Macrovipera lebetina cernovi* (Kazakstan), *Macrovipera lebetina obtusa* (Azerbaijan), *Macrovipera lebetina turanica* (Turkmenistan), *M. schweize*ri (Greece), *Montivipera albizona* (Turkey), *Montivipera bornmuelleri* (Israel), *Montivipera bulgardaghica* (Turkey), *Montivipera raddei*, *Montivipera latiffi* (Iran), *Montivipera wagneri* (Turkey), *M. xanthina* (Turkey), *V. ammodytes* (Ada Island, Montenegro), *V. a. meridionalis* (Greece), *V. a. montandoni* (Bulgaria), *V. aspis aspis* (France), *V. aspis hugyi* (Italy), *V. berus* (Norway), *V. kaznakovi* (Turkey), *V. latastei latastei* (Burgos, Spain), *V. latastei gaditana* (Spain), *V. nikolskii* (Russia), *V. renardi* (Russia), and *V. transcaucasiana* (Turkey)*.*

All venom work was undertaken under the auspices of UQ biosafety approval #IBC134BSBS2015 and UQ animal ethics approval # 2021/AE000075.

### Mimotope Production and Preparation

Thirteen–fourteen amino acid chain mimotope of the nAChR orthosteric site of vertebrate α-1 nAChR subunit was designed from publicly available sequences of cholinergic receptors (Chrna1) from Genbank and UniProt (amphibian α1 (WVYYDSSPETPYLD designed from uniprot F6RLA9), lizard α1 (WVVYASSTETPYLD from Genbank XM_015426640), avian α1 (WVYYASSPDTPYLD designed from uniprot E1BT92), rodent α-1 (WVFYSSSPNTPYLD designed from uniprot P25108), and human α-1 (SVTYSSSPDTPYLD designed from uniprot G5E9G9)) and was developed by GenicBio Ltd. (Shanghai, China). Comparisons between whole receptor and nAChR mimotopes testing using kinetics data should be approached with caution as Ser-Ser was replaced for Cys-Cys of the native mimotope to avoid uncontrolled postsynthetic thiol oxidation. Though this change does not cause any effect as Cys-Cys does not take part in the analyte binding. Biotin linker bound to two aminohexanoic acid (Ahx) spacers were synthesized with the mimotopes forming a 30 Å linker. Dry stocks of the mimotopes were solubilized with Dimethyl sulfoxide (DMSO) and diluted to 1/10 dilution to produce a working stock of 50 µg/ ml.

### Bio-Layer Interferometry (BLI) Assay

To check affinity on nicotinic receptor subunit α 1, bio-layer interferometry (BLI) assay was performed using the Octet HTX system (Sartorius, Germany) (Zdenek et al. 2019; Harris et al. 2020a; b; Youngman et al. 2021). Streptavidin-coated biosensors with immobilized biotinylated amino acids (mimotopes) are dipped and equilibrated in the buffer, followed by exposure to the venom. If the venom has toxins targeting the receptor (amino acids mimotopes), they will attach and accumulate. This sends signals in waves of light due to association. The more accumulation of analytes to the ligand, the larger the wavelength shifts. This accurate measuring of the wavelength shift from the baseline compared to once the toxins have bound to their receptor will allow us to evaluate the binding speed and strength of this biomolecular interaction. The above process was repeated, (1) replacing mimotopes with buffer, (2) replacing venom with buffer, and (3) replacing both mimotopes and venom with buffers, as controls to nullify any possibility of non-specific bindings. The methodology and data analysis were based upon the validated protocol of Zdenek et al. (2019) and Harris et al. (2020a, b, c).

Before starting the experiment, the Streptavidin biosensors were hydrated in assay running buffer (1 × Dulbecco’s phosphate-buffered saline (DPBS) with 0.1% Bovine serum albumon (BSA) and 0.05% Tween-20) for 30 min and agitated on a shaker. The working stock of mimotopes (50 µg/ml) was further diluted to an experimental concentration of 1 µg/ml per well and venom to 50 µg/ml. Standard acidic solution (glycine buffer- 10 mM glycine (pH 1.5–1.7) in ddH_2_O) was used for the analyte (venom) dissociation step. All the steps were done in a set of triplicates. Data of the association step was obtained from the Octet HTX system in excel format and then input into Prism 8.0 software (GraphPad Software Inc., La Jolla, CA, USA) to generate area under curve (AUC) values and later to the graph.

## Results and Discussion

**Fig. 1:**
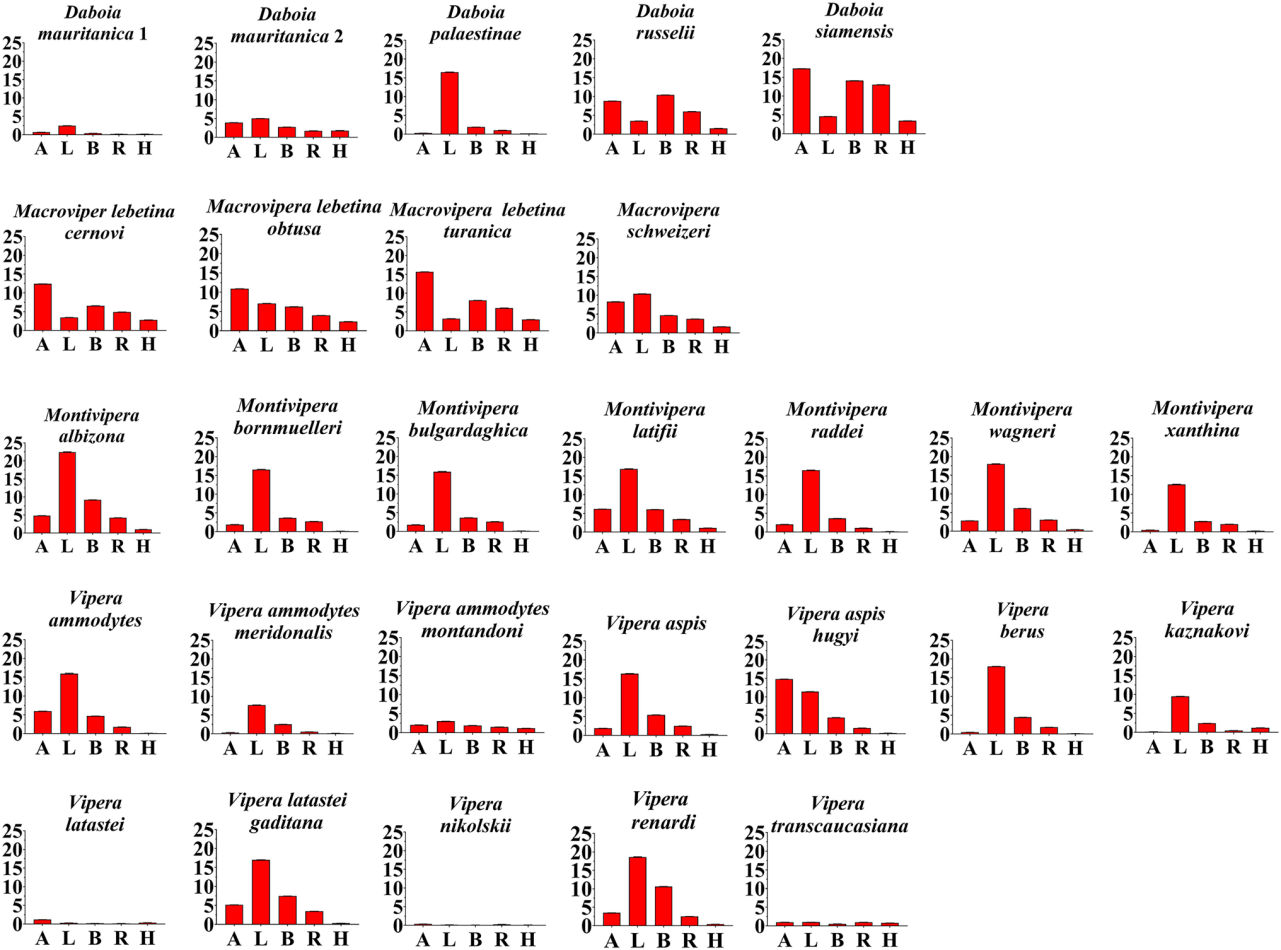
Bar graphs with AUC, showing relative neurotoxic potency of Palearctic vipers towards α-1 nAChR mimotopes from various taxa. A = amphibian, L = lizard, B = bird, R = rodent, and H = human. Higher AUC values indicate higher potency. Y- axis values are AUC (area under the curve) N = 3 mean ± standard deviation. Scales are the same for each graph to make the relative potency readily apparent. See Fig. 2 for depictions of relative potency within a target and see Table 1 for values for each species/mimotope combination

There were no non-specific binding observed with the sensors in any of the cases of controls mentioned above. Interpretation of binding to the different targets must be made from two different points of view. First, from the perspective of the overall AUC values. We observed a wide degree of binding affinity to each target by all the venoms, with lizard having the highest AUC (22.4 ± 0.124 for *Montivipera albizona*), while the highest amphibian targeting was 17.3 ± 0.01 by *D. siamensis*, bird 14.1 ± 0.019 by *D. siamensis*, rodent 13 ± 0.023 by *D. siamensis*, and human 3.42 ± 0.015 by *D. siamensis* venom (Table 1)*.* According to AUC values, for most venoms that displayed neurotoxicity, the lizard target was the most potently targeted (Fig. 1). The trend of venoms displaying the greatest binding potency toward a particular taxon (lizard) is not unique to the venoms in this study; Cobras in the genus *Naja* bound the amphibian target the strongest (Harris et al. 2020c), as did Asian tree vipers in the *Tropidolaemus* genus (Harris et al. 2020b), while large African vipers such as the Gaboon viper (*Bitis gabonica*) bound to the bird mimotope the strongest (Youngman et al. 2021). Broad potency across multiple taxa has also been observed: venom from the death adder *Acanthophis antarcticus* exhibited nearly equipotent binding across all the targets (Zdenek et al. 2019). In contrast, the lowest affinity for the human target is a general trend observed for other venoms, whereby Afro-Asian primates evolved resistance to the venoms of sympatric cobras and that within the Afro-Asian primates, the chimpanzee/gorilla/human last common ancestor potentiated this resistance to alpha-neurotoxins (Harris et al. 2021), as part of a broader reciprocal chemical-arms race (Kazandjian et al. 2021). The relatively strong binding by *Daboia* species is, however, consistent with clinical reports of neurotoxic human envenomations (Silva et al. 2016). In contrast, the results of this study suggest that the neurotoxicity symptoms seen in *V. ammodytes* and *V. aspis* envenomations (de Haro et al. 2002; Ferquel et al. 2007; Logonder et al. 2008; Malina et al. 2013; Varga et al. 2018) are not due to binding to the alpha-1 orthosteric site but due either to binding to the alpha-1 allosteric site or presynaptically binding.

**Table 1:** Areas under the curve for binding to alpha-1 orthosteric site mimotopes. Values are N = 3 mean + / − standard deviation

**Palearctic viper species**	**Mimotopes**				
	**Amphibian**	**Lizard**	**Bird**	**Rodent**	**Human**
***Daboia mauritanica* 1**	0.7 ± 0.006	2.46 ± 0.016	0.415 ± 0.011	0.177 ± 0.006	0.185 ± 0.009
***D. mauritanica* 2**	3.91 ± 0.011	5.02 ± 0.009	2.75 ± 0.016	1.73 ± 0.028	1.77 ± 0.074
***D. palaestinae***	0.289 ± 0.007	16.5 ± 0.074	1.9 ± 0.006	1.05 ± 0.014	0.18 ± 0.003
***D. russelii***	8.8 ± 0.016	3.5 ± 0.01	10.4 ± 0.026	6.01 ± 0.014	1.52 ± 0.021
***D. siamensis***	17.3 ± 0.01	4.6 ± 0.008	14.1 ± 0.019	13 ± 0.023	3.42 ± 0.015
***Macrovipera lebetina cernovi***	12.4 ± 0.014	3.46 ± 0.011	6.56 ± 0.04	4.9 ± 0.021	2.8 ± 0.008
***M. lebetina obtuse***	10.9 ± 0.024	7.1 ± 0.021	6.24 ± 0.028	3.99 ± 0.02	2.4 ± 0.025
***M. lebetina turanica***	15.7 ± 0.015	3.25 ± 0.01	8.1 ± 0.017	6.07 ± 0.026	3.02 ± 0.01
***M. schweizeri***	8.3 ± 0.026	10.4 ± 0.032	4.65 ± 0.024	3.72 ± 0.017	1.66 ± 0.018
***Montivipera albizona***	4.82 ± 0.021	22.4 ± 0.124	9.18 ± 0.039	4.23 ± 0.034	1.02 ± 0.006
***M. bornmuelleri***	1.88 ± 0.018	16.5 ± 0.09	3.65 ± 0.022	2.71 ± 0.017	0.118 ± 0.018
***M. bulgardaghica***	1.77 ± 0.03	15.9 ± 0.113	3.64 ± 0.035	2.61 ± 0.027	0.122 ± 0.013
***M. latifii***	6.18 ± 0.007	16.9 ± 0.069	6.06 ± 0.014	3.43 ± 0.014	1.11 ± 0.018
***M. raddei***	2.04 ± 0.022	16.5 ± 0.088	3.63 ± 0.026	1.07 ± 0.044	0.152 ± 0.016
***M. wagneri***	2.83 ± 0.008	18 ± 0.09	6.13 ± 0.032	3.05 ± 0.021	0.483 ± 0.006
***M. xanthina***	0.479 ± 0.024	12.6 ± 0.099	2.78 ± 0.022	2.03 ± 0.019	0.229 ± 0.011
***Vipera ammodytes***	5.96 ± 0.018	15.9 ± 0.17	4.66 ± 0.023	1.75 ± 0.017	0.104 ± 0.007
***V. ammodytes meridonalis***	0.346 ± 0.005	7.66 ± 0.065	2.52 ± 0.019	0.532 ± 0.013	0.154 ± 0.013
***V. ammodytes montandoni***	2.07 ± 0.02	3.02 ± 0.025	1.9 ± 0.012	1.52 ± 0.012	1.19 ± 0.01
***V. aspis***	1.91 ± 0.01	16.3 ± 0.081	5.42 ± 0.026	2.51 ± 0.007	0.28 ± 0.005
***V. aspis hugyi***	14.8 ± 0.035	11.4 ± 0.036	4.38 ± 0.023	1.58 ± 0.005	0.202 ± 0.011
***V. berus***	0.464 ± 0.013	18 ± 0.043	4.45 ± 0.018	1.8 ± 0.009	0.12 ± 0.004
***V. kaznakovi***	0.182 ± 0.012	9.47 ± 0.045	2.4 ± 0.016	0.488 ± 0.013	1.23 ± 0.009
***V. latastei***	1.18 ± 0.009	0.3 ± 0.012	0.155 ± 0.007	0.142 ± 0.013	0.365 ± 0.01
***V. latastei gaditana***	5.16 ± 0.008	17 ± 0.056	7.46 ± 0.024	3.47 ± 0.011	0.308 ± 0.006
***V. nikolskii***	0.39 ± 0.008	0.165 ± 0.01	0.114 ± 0.004	0.282 ± 0.004	0.131 ± 0.006
***V. renardi***	3.48 ± 0.013	18.6 ± 0.098	10.6 ± 0.052	2.49 ± 0.019	0.4 ± 0.009
***V. transcaucasiana***	0.987 ± 0.005	0.989 ± 0.009	0.529 ± 0.023	0.981 ± 0.007	0.796 ± 0.008

**Fig. 2:**
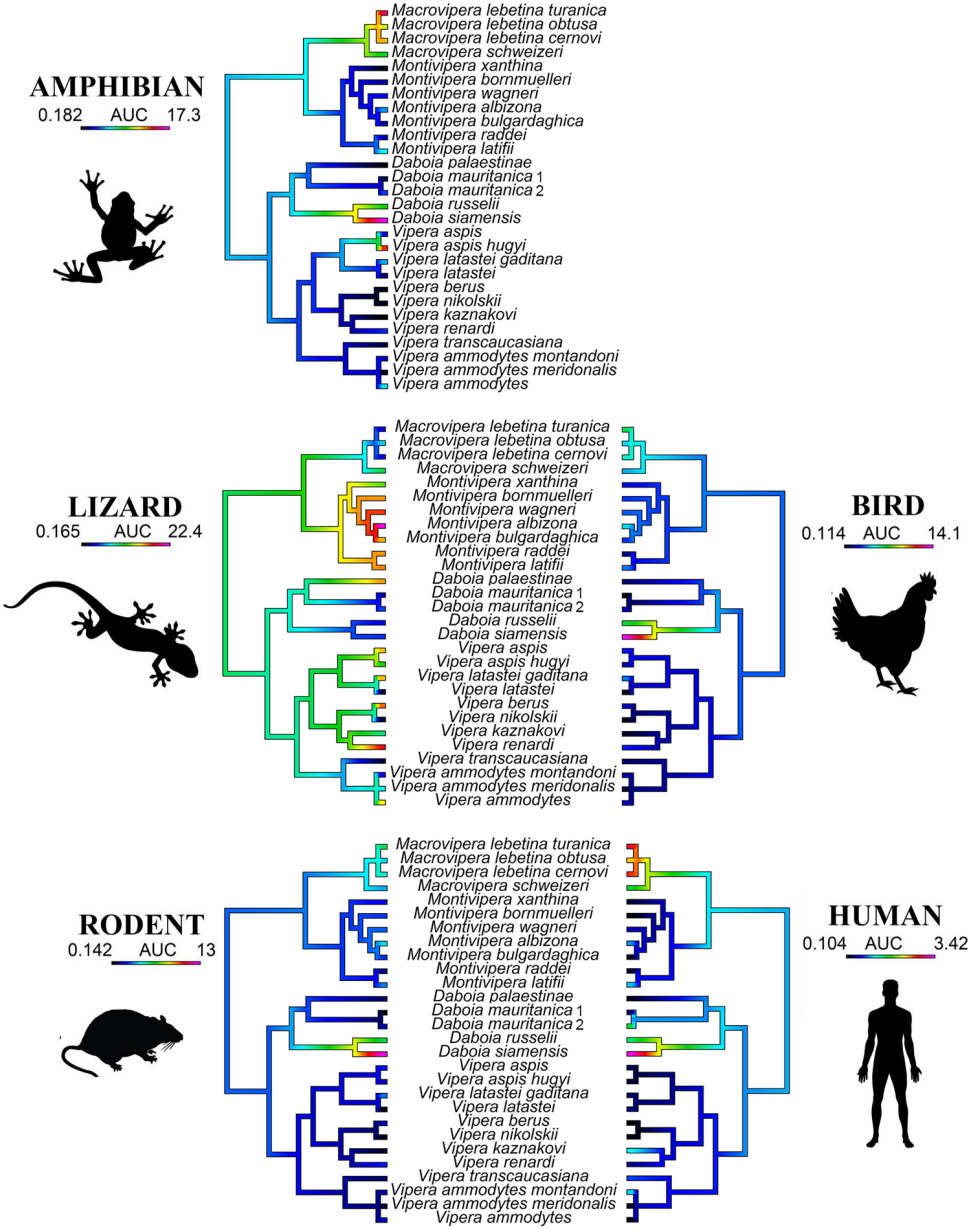
Ancestral reconstruction of relative neurtoxicity mapped over the Paleactic viperid snake organismal tree. Warmer colors correspond to higher AUC values (greater potency) while cooler colors correspond to lower AUC values (lesser potency). Note that each target organism is scaled for relative effect within that organism, not for relative effect between organisms. Thus, overall potency varies between organisms, with lizard the most strongly bound and human the least. See Fig. 1 for graphing of potency using the same scale for all targets and see Table 1 for values for each species/mimotope combination. Phylogeny based upon Freitas et al. (2020)

The second point of interpretation is from the perspective of how the trends of relative potency within each target compared against the other targets (Fig. 2). In other words, was the most relatively potent for a particular mimotope also the most potent against the other mimotopes? This was clearly the case for *D. siamensis* which was the most potent against all the mimotopes except for lizard, where its relative potency was much less that of other species (3.5 ± 0.01 versus 22.4 ± 0.124 for *Montivipera albizona*). Similarly, the *Montivipera* species were the most potent on lizard, but had negligible activity upon other targets. And within the *Vipera* genus, *V. ammodytes meridonalis*, *V. aspis*, *V. berus*, *V. kaznakovi*, *V. latastei gaditana*, and *V. renardi* demonstrated strong binding to lizard, while other targets bound at much lower relative levels. In contrast, while *V. aspis hugyi* binds lizard at high levels, it was unusual among *Vipera* species, binding to the amphibian target even more potent.

Conversely, *Macrovipera* species bound the amphibian targets most strongly relative to the other targets, except for *M. schweitzeri* which had relative binding stronger to the lizard than the amphibian target. The *M. lebetina* subspecies also had the strongest relative binding to the human target, which was exceeded only by *D. siamensis.* Significant variation of relative potency across targets has been noted for other species, where the most potent for one target was not correspondingly the most potent across all targets (Harris et al. 2020a, b, c; Youngman et al. 2021, 2022; Zdenek et al. 2019). This is consistent with evolutionary selection pressures shaping affinities for particular targets.

In conclusion, the results of this study shed light on the diversity of neurotoxic venoms within this clade of fascinating snakes. This shapes our understanding of the evolutionary selection pressures acting upon these venoms by providing indications of prey-lineage selective effects. In contrast, the data also reveals that relying upon human case reports to provide indications of neurotoxic species may obscure species potent against non-human animals but with little effect upon humans. The extremely dynamic variation also suggests multiple cases of amplification of this trait and cases of secondary loss. In a previous study, the *Montivipera species*, which have evolved to be high-altitude specialists, were unique in having switched from the Factor X-activating procoagulant venom phenotype, a basal trait of the clade, to a Factor Xa inhibiting anticoagulant venom phenotype (Chowdhury et al. 2021). In this study, this genus was unique in being the only one that was consistently potently active upon the lizard alpha-1 orthosteric site. Similarly, *V. latastei gaditana* has evolved to be a high-altitude specialist and has secondarily lost the procoagulant trait (Chowdhury et al. 2021). Moreover, *V. latastei gaditana* was also potently neurotoxic on lizards, unlike the low-altitude *V. latastei*, which was devoid of neurotoxicity but was shown previously to be potently procoagulant (Chowdhury et al. 2021). Within the *Vipera*, the species devoid of neurotoxic action (*V. latasti*, *V*. *nikolskii*, and *V. transcaucasiana*) or extremely low (*V. ammodytes montandoni*) were not each other’s closest relatives, with each of these being sister species to potently neurotoxic species. As each of these are not basal species either, this leads to two competing hypotheses: that the *Vipera*’s last common ancestor was weakly neurotoxic and that there were multiple independent cases of amplification of this shared trait or that the last common ancestor was potently neurotoxic and there were multiple cases of secondary loss. Relative to all other *Daboia*, *D. palestinae* is unique in upregulating the binding to the lizard alpha-1 orthosteric site, while the last common ancestor of *D. russelii/siamensis* upregulated the binding to other taxonomical lineages*.* The last common ancestor of *Macrovipera* upregulated binding to the amphibian alpha-1 orthosteric site. These dynamic variations underscore what a labial trait venom is and reinforce the usefulness of high-throughput assay techniques that facilitate the investigation of large numbers of samples to reconstruct such complex evolutionary histories.

## Data Availability

All the data used in the manuscripts is available in Table 1.
